# A methodology for incorporating a photon‐counting CT system into routine clinical use

**DOI:** 10.1002/acm2.14069

**Published:** 2023-06-30

**Authors:** Steven T. Bache, Ehsan Samei

**Affiliations:** ^1^ Department of Radiology Clinical Imaging Physics Group Duke University Medical Center Durham North Carolina USA; ^2^ Carl E. Ravin Advanced Imaging Laboratories Duke University Medical Center Durham North Carolina USA

**Keywords:** CT image quality, CT protocols, photon‐counting CT

## Abstract

Photon‐counting computed tomography (PCCT) systems are increasingly available in the U.S. following Food and Drug Administration (FDA) approval of the first clinical PCCT system in Fall 2021. Consequently, there will be a need to incorporate PCCTs into existing fleets of traditional CT systems. The commissioning process of a PCCT was devised by evaluating the degree of agreement between the performance of the PCCT and that of established clinical CT systems. A PCCT system (Siemens NAEOTOM Alpha) was evaluated using the American College of Radiology(ACR) CT phantom (Gammex 464). The phantom was scanned on the system and on a 3rd Generation EID CT system (Siemens Force) at three clinical dose levels. Images were reconstructed across the range of available reconstruction kernels and Iterative Reconstruction (IR) strengths. Two image quality metrics—spatial resolution and noise texture—were calculated using AAPM TG233 software (imQuest), as well as a dose metric to achieve target image noise magnitude of 10 HU. For each pair of EID‐PCCT kernel/IR strengths, the difference in metrics were calculated, weighted, and multiplied over all metrics to determine the concordance between systems. IR performance was characterized by comparing relative noise texture and reference dose as a function of IR strength for each system. In general, as kernel “sharpness” increased for each system, spatial resolution, noise spatial frequency, and reference dose increased. For a given kernel, EID reconstruction showed higher spatial resolution compared to PCCT in standard resolution mode. PCCT implementation of IR better preserved noise texture across all strengths compared to the EID, demonstrated by respective 20 and 7% shifts in noise texture from IR “Off” to IR “Max.” Overall, the closest match for a given EID reconstruction kernel/IR strength was identified as a PCCT kernel with “sharpness” increased by 1 step and IR strength increased by 1–2 steps. Substantial dose reduction potential of up to 70% was found when targeting a constant noise magnitude.

## 1 INTRODUCTION

With the recent FDA clearance of the first commercial Photon‐counting computed tomography (PCCT) system,^1^ there will undoubtedly be a surge in interest in bringing this cutting‐edge technology into routine clinical use. PCCT detectors differ from traditional energy integrating detectors (EID) by capturing energy information from each individual photon that reaches the detector, allowing energy‐weighting, a decrease in electronic noise, and availability of spectral information without the use of dual energy acquisitions.^2^, ^3^, ^4^ PCCT detector technology promises several advantages over traditional EID CT systems, including increased spatial resolution, lower noise, more accurate quantification, increased iodine contrast‐to‐noise ratio, all at potentially lower radiation doses.^3^, ^5^, ^6^, ^7^, ^8^, ^9^, ^10^, ^11^, ^12^, ^13^, ^14^, ^15^

While PCCT technology shows great potential in improving the overall diagnostic quality of CT examinations, there remains a practical issue of incorporating such a system into routine clinical use where it is likely that one or more EID CT systems are currently producing satisfactory images at appropriate radiation doses. This work aims to outline a methodology for translating and relating image quality metrics from existing EID CT images into suitable image perception on the PCCT, representing a key first step in utilizing a PCCT system into a clinical practice. The goal was not necessarily to match the PCCT performance to EID, rather to develop insight about the differences based on common metrics to enable informed decision for best integration into practice.

## 2 METHODS

Axial images of the American College of Radiology (ACR) CT accreditation phantom (Gammex 464, Gammex, Inc., Middleton, WI, USA) were acquired using a traditional EID CT scanner (Siemens SOMATOM Force) and a PCCT scanner (Siemens NAEOTOM Alpha, Siemens Healthcare, Germany) at two clinically appropriate dose levels. Images were reconstructed using routine abdominopelvis slice thickness using several convolution kernels and Iterative reconstruction (IR) strengths. PCCT images were reconstructed as 70 keV virtual monoenergetic image (VMI) sets, while EID images were conventional full polyenergetic spectrum images. Table 1 shows acquisition and reconstruction settings for all images.

**TABLE 1:** Phantom acquisition and reconstruction parameters.

Parameter	Siemens SOMATOM force	Siemens Naeotom alpha
Detector type	Energy‐integrating detector (EID)	Photon‐Counting (PC)
CTIDvol (mGy, 32 cm)	4.98, 15.0, 30.0	5.0, 15.0, 25.0
Spiral pitch factor	1	1
Detector configuration	96 × 0.6 mm (57.6 mm)	144 × 0.4 mm (57.6 mm)
Reconstructed FOV (mm)	220	220
Slice thickness (mm)	5	5
	Body	Body
	Bf: 36, 40, 44	
	Bl: 57, 64	Bl: 56, 60, 64
	Br: 32, 36, 40, 44, 49, 54, 59, 64, 69	Br: 36, 40, 44, 48, 56, 64, 72
	Bv: 36, 40, 44, 49, 59	Bv: 36, 44, 56
	Head	Head
	Hc: 40, 44	Hc: 40, 44
Convolution kernels	Hf: 38, 40	
	Hp: 38	
	Hr: 32, 36, 38, 40, 44, 49, 54, 59, 64, 69	Hr: 36, 40, 44, 48, 56, 64, 72
	Ht: 39, 43	
		Hv: 36, 44, 56
	Quantitative	Quantitative
	Qr: 32, 36, 40, 44, 49, 54, 59, 69, 70	Qr: 36, 40, 44, 48, 60, 68, 76
Reconstruction types	FBP, ADMIRE	FBP, QIR
Spectral recons	Full spectrum polyenergetic	70 keV Monoenergetic
IR strengths	0, 1, 2, 3, 4, 5	0, 2, 4

### 2.1 Image perception and dose metrics

All analysis was performed using an open‐source MATLAB software package (imQuest, Duke University).^16^ The software calculates all metrics according to AAPM Task Group 233 methodology.^17^ Measurements of noise texture, noise magnitude, and spatial resolution were made from each CT reconstruction.

For each image series, the Task Transfer Function (TTF) was calculated from the ACR phantom Module 1 “Air” insert.^18^, ^19^, ^20^ A scalar representation of TTF, *f_50_*, defined as the spatial frequency at which the system response is reduced by 50%, was computed

In addition, 2D Noise Power Spectrum (NPS) was from each reconstructed image set was measured by taking the Fourier Transform of four 30  × 30 mm ROIs within the uniform water section of the ACR phantom. The 2D NPS was radially averaged to estimate the 1D frequency distribution of noise for the given reconstruction kernel and IR strength. To represent noise texture as a scalar metric the average NPS frequency, *f_av_*, was calculated as

(1) $$fav=\frac{\;\sum_{i}f_{i}w_{i}}{\sum_{i}w_{i}}\;$$

**FIGURE 1:**
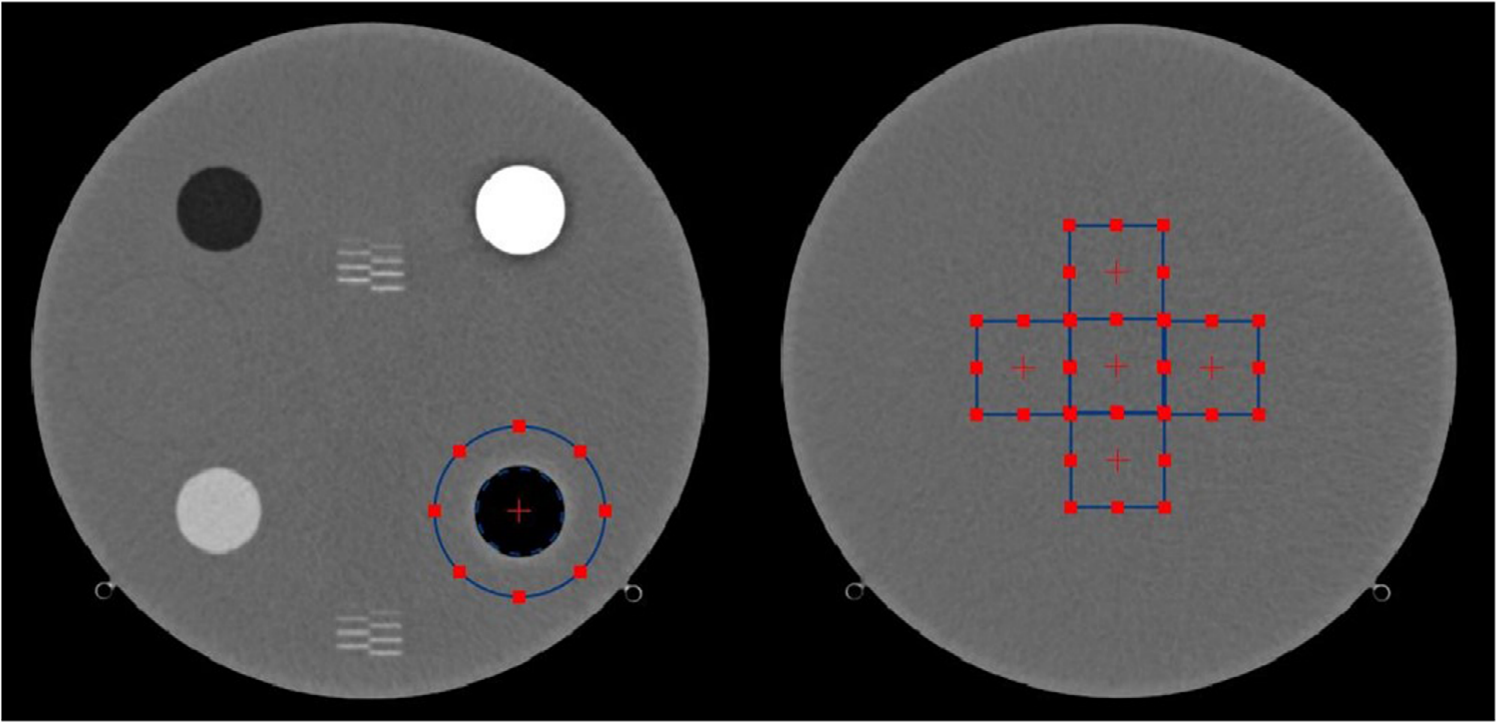
ROI placement for measuring Task transfer function (TTF, Left), Noise power spectrum (NPS), and noise magnitude (Right). TTF, Task transfer function; NPS, Noise power spectrum.

Where w_i_ is the NPS magnitude at frequency *f*_i_,.^16^ Figure 1 shows the regions of interest used to compute TTF and NPS.

Noise magnitude, or CT number standard deviation, was calculated using the same four ROIs used for NPS calculation as outlined above. A power‐law relationship was developed between noise magnitude and radiation dose level for each scanner/kernel/IR strength of the form

(2) $$N=a\times d^{b},$$

Where N is noise magnitude, d is reported radiation dose level in mGy CTDIvol for the 32 cm phantom, and a and b are fitting parameters. This relationship was utilized to determine a scalar radiation dose metric *d_ref_*, defined as the radiation dose for each reconstruction type in mGy required to obtain a noise magnitude of 10 HU.

### 2.2 Perception matching methodology

The methodology for determining best perception “matches” between the EID and PCCT systems closely followed the methods described in Winslow et al.^21^ First, relative difference between PCCT with respect to EID were computed for spatial resolution metric *f_50_*, noise texture metric *f_av_*, and reference dose metric *d_ref_* for all reconstructions. Relative difference magnitude and sign were preserved, as the matching algorithm detailed in the next section required distinction between whether spatial resolution and reference dose were greater or lesser for the PCCT compared to the EID system.

To determine the degree to which reconstructions matched between the PCCT and EID system, the relative difference matrices were passed through metric‐specific sigmoid weighting functions of the form

(3) $$w_{x}\left(x,a_{x},c_{x}\right)\;=\frac{1}{1+\;e^{-a_{x}\left(x-c_{x}\right)}}\;,$$

where x is the relative difference matrix for a specific metric, *a*_x_ and *c*_x_ are sigmoid function parameters, and w_x_ is the weighting function for that metric. Parameter *c* determines the value at which the weighting function equals 0.5, while parameter *a* determines how steeply the weighting function crosses the *c* threshold. A perfect match for a particular metric x occurs when *x* = 0 and *w*_x_ = 1. Sigmoid parameters used for this work were adapted from the “Dose‐Conscious” parameters from Winslow et al.^21^ reproduced in Table 2.

**TABLE 2:** Weighting function parameters and form used for calculating degree by which reconstructions are “matched”, given the relative difference in three image quality metrics.

Metric	Input (x)	a_x_	c_x_	Output	Functional form
Resolution	*f50_p,e_*	150	−0.05	*R*	R_p,e_ = w_r_(r_p,e_,a,c)
Texture	*fav_p,e_*	150	−0.05	*T*	T_p,e_ = w_t_(t_p,e_,a,c)—w_t_(t_p,e_,a,‐c)
Dose	*dref_p,e_*	15	−0.33	*D*	D_p,e_ = w_r_(‐d_p,e_,a,c)

Abbreviation: EID, Energy integrating detectors; FBP, Filtered back‐projection; IR, Iterative reconstruction; PCCT, Photon‐counting computed tomography.

**FIGURE 2:**
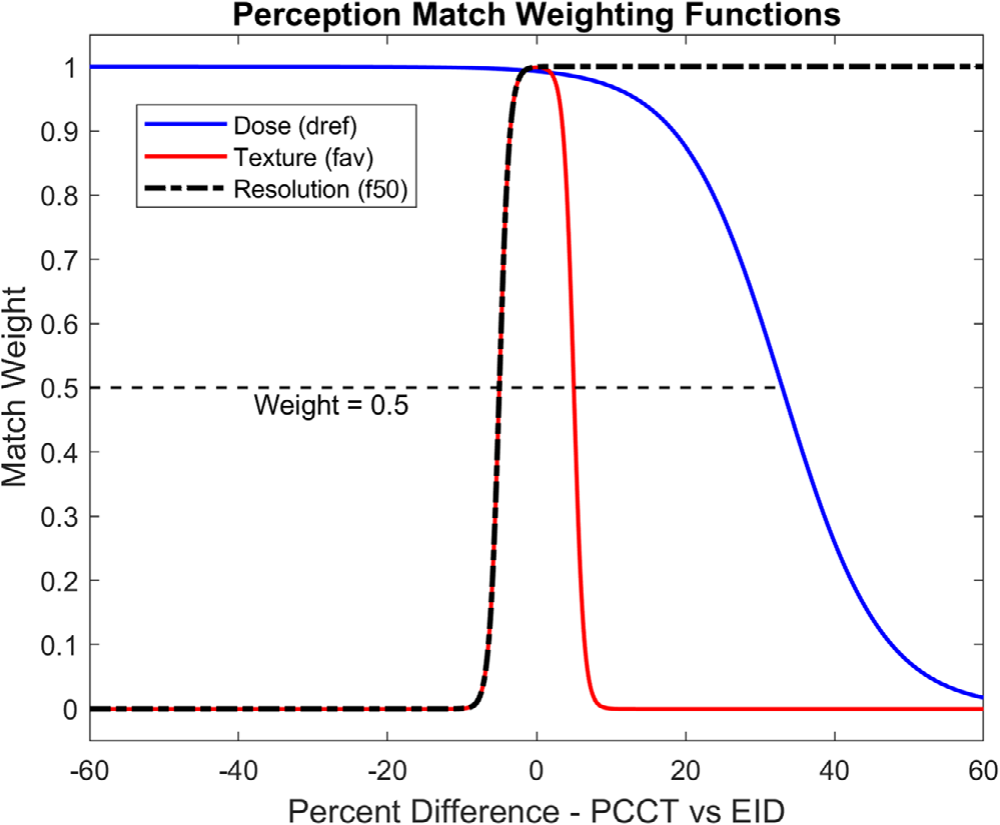
Sigmoid weighting functions for computing similarities in dose, noise texture, and spatial resolution metrics for EID and PCCT reconstruction/kernel combinations. EID, Energy integrating detectors; PCCT, Photon‐counting computed tomography.

The parameters of the resolution sigmoid weighting function were chosen such that a PCCT reconstruction was given maximum weight (*R* = 1) at equal or greater resolution, half weight (*R* = 0.5) at 5% reduced resolution, down to virtually zero at 10% reduced resolution. Texture sigmoid function parameters were selected to exhibit two‐sided weighting, with half weight given when PCCT *f_av_* values fell outside ± 5% of the compared EID reconstruction. Dose sigmoid weighting function were selected to give a similar one‐sided weighting as the resolution function, but with a half weight threshold at 33% increased dose for the PCCT compared to an EID reconstruction. The rationale for these parameters mirror Winslow et al,^21^ with the exception that the 0.5 weighting penalty for increased dose was shifted from 50% increased dose down to 33%, in line with the expectation that the new PCCT technology should have better radiation dose performance. Consequently, a PCCT reconstruction was considered closely matched (*R, T*, or *D* ≥ 0.9) to an EID reconstruction if its spatial resolution in terms of *f_50_* was not less than 3.5% lower, it's noise texture in terms of *f_av_* was within ± 3.5%, and its reference dose *d_ref_* was no more than 17% greater. Figure 2 shows the functional form of each sigmoid weighting factor.

How closely matched a PCCT and EID reconstruction were, M, was calculated as the product of the weights determined for each of the three metrics, or

(4) $$M_{p,e}=\;R_{p,e}\times T_{p,e}\times D_{p,e}.$$

For each of the three metrics, an ideal match resulted in a weighted relative difference of 1.0, thus an

ideal overall match for a given EID reconstruction kernel/IR strength resulted in an overall match, *M*, of 1.0.

### 2.3 IR characterization

In addition to identifying best PCCT reconstruction kernel/IR strengths for given EID kernels, the calculated metrics were used to characterize the overall influence of IR strength on noise texture (*f_av_*) and noise magnitude. Reconstructions using iterative and/or model‐based techniques have a tendency to shift the spatial frequency components of image noise towards lower frequencies (often referred to as “blurred”, “plastic”, etc.), which can affect both image perception and overall diagnostic confidence from a given image series.^22^ The Siemens NAEOTOM Alpha IR algorithm (Quantum Iterative Reconstruction, or QIR) is specifically tailored to the spectral complexity of PCCT data.^23^, ^24^ Noise texture and noise magnitude were analyzed as a function of IR strength for both PCCT (QIR) and EID (Advanced Modeled Iterative Reconstruction, or ADMIRE) algorithms and for several common kernels.

## 3 RESULTS

### 3.1 Image perception and dose metrics

**FIGURE 3:**
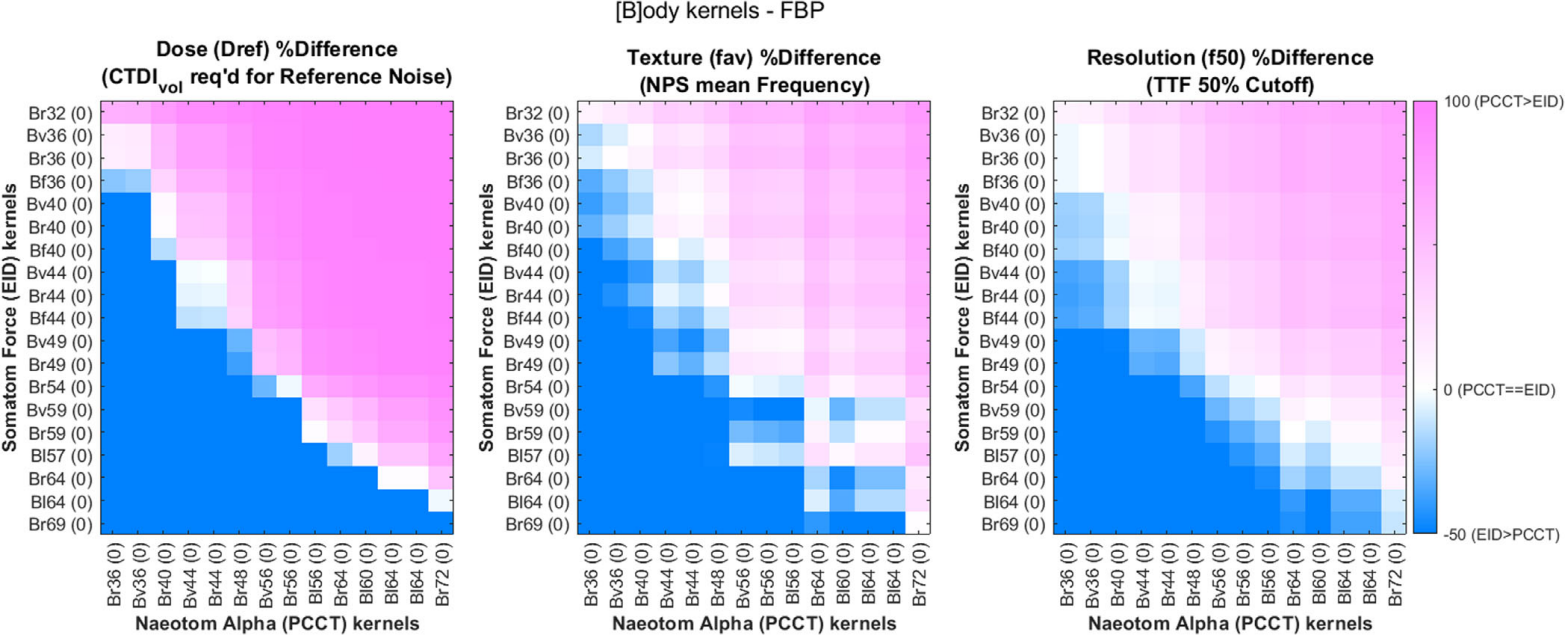
Relative differences in image perception metrics between all Body kernels with filtered back‐projection reconstruction on EID and PCCT systems, computed relative to EID kernels. FBP and IR results can be found in supplemental figures. Both axes are sorted by reference Dose (dref) in all plots for ease of presentation. Head and quantitative kernels showed similar results and can be found in supplemental figures. EID, energy integrating detectors; PCCT, Photon‐counting computed tomography; IR, Iterative Reconstruction.

Figure 3 shows representative percent differences results for all three metrics for Body reconstruction kernels, while Head and Quantitative kernels showed similar results. As expected, an increase in reconstruction kernel sharpness improved spatial resolution (*f_50_*) and increased the spatial frequency component of noise texture (*f_av_*), with a penalty of increased reference dose (*d_ref_*). This trend is demonstrated by comparing relative sharp kernels from one system to relative smooth kernels on the other in Figure 3.

### 3.2 Reconstruction kernel perception matches

**FIGURE 4:**
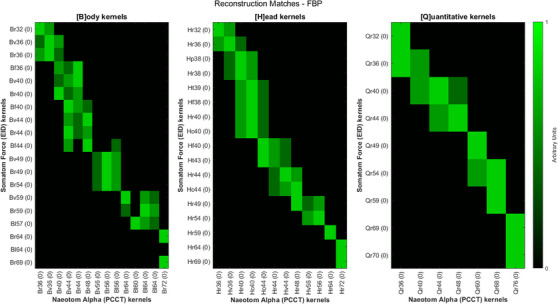
Final reconstruction match values for all kernels with filtered back‐projection (FBP) reconstruction. Matches for IR and FBP can be found in supplemental Figures. A value of 1 indicates that a particular PCCT kernel/IR combination is the best possible match for a given EID kernel/IR combination.EID, energy integrating detectors; FBP, filtered back‐projection; IR, Iterative reconstruction; PCCT, Photon‐counting computed tomography.

Figure 4 shows the final “Match” value between each kernel with filtered back‐projection (no IR), with a value of 1.0 indicating the best possible match between scanner types. Match results for all kernels and IR strengths can be found in the supplemental materials. In general, the best match for a given EID kernel/IR strength was either an identically named PCCT kernel or, more often, a kernel increased by one nominal sharpness “step” and with IR strength increased by 1−2 steps.

**FIGURE 5:**
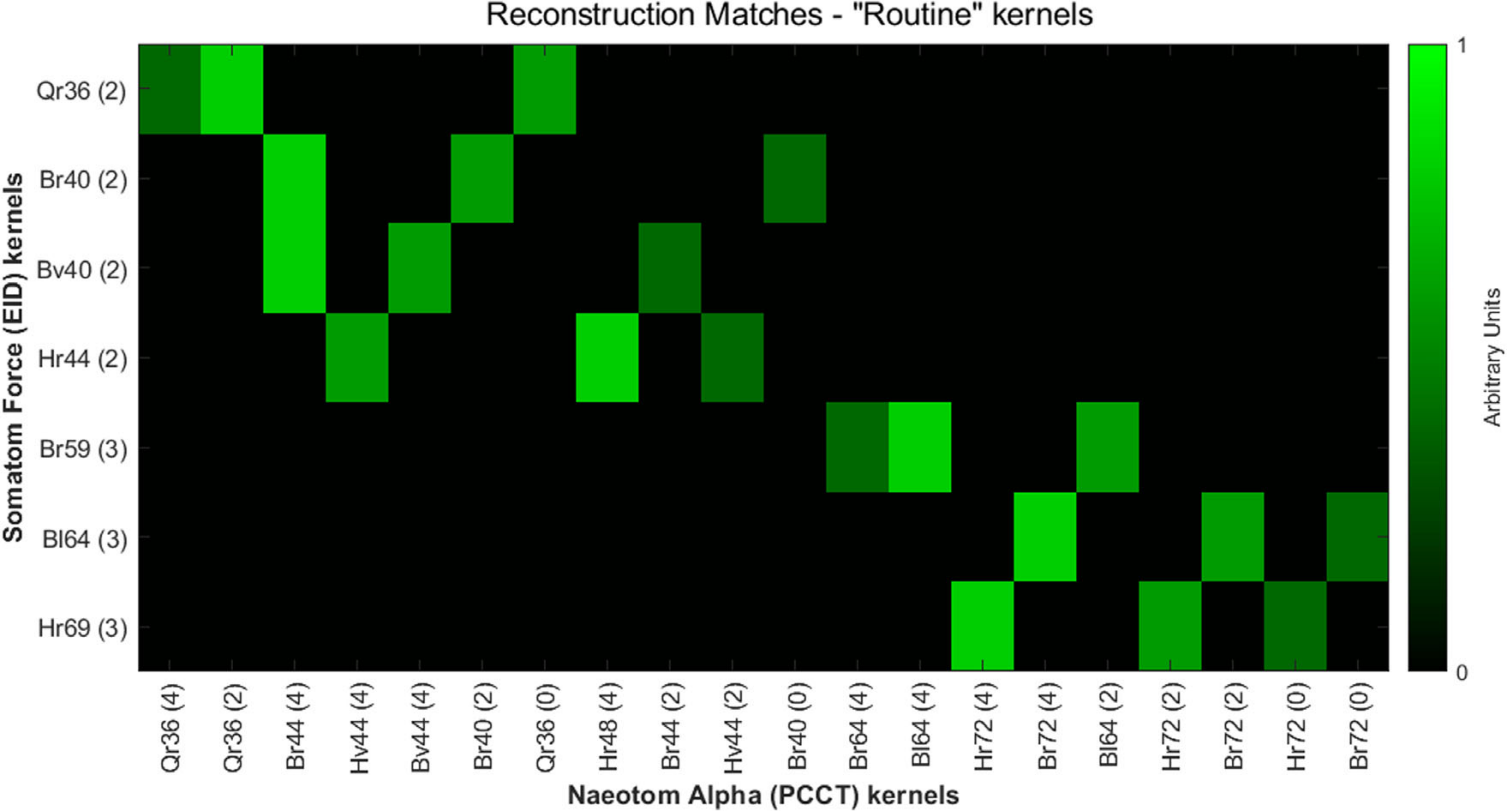
Best PCCT kernel matches for routine brain, chest, and abdomen kernels used at our institution.

At our institution, the most common reconstruction kernels (and use) are Br40 (soft tissue body), Br59 (sharp/bone body), Bv40 (vascular body), Bl64 (high resolution lung), Hr44 (soft tissue head), Hr69 (sharp/bone head), and Qr36 (quantitation), with IR routinely set to mid‐range strengths of 2 for soft tissue and 3 for sharper kernels . Results in Figure 4 are condensed to show the top three matches for only these kernels from Figure 5. Results for these specific kernels are also tabulated in Table 3, as well as dose differences for each best‐matched PCCT kernel compared to target EID kernel.

**TABLE 3:** Best PCCT reconstruction kernel (IR strength) matches for each “routine” EID reconstruction kernel (IR strength). Dose differences are relative to target EID kernel.

PCCT Top 2 Matches—kernel (IR Strength)				
EID Target kernel (IR)	First	Δd_ref_ (%)	Second	Δd_ref_ (%)
Qr36 (2)	Qr36 (2)	–19	Qr36 (0)	59
Br40 (2)	Br44 (4)	–43	Br40 (2)	–26
Bv40 (2)	Br44 (4)	–37	Bv44 (4)	–28
Hr44 (2)	Hr48 (4)	–51	Hv44 (4)	–74
Br59 (3)	Bl64 (4)	50	Br64 (4)	–10
Bl64 (3)	Br72 (4)	–30	Br72 (2)	53
Hr69 (3)	Hr72 (4)	–56	Hr72 (2)	–2

*Note*: Δ values refer to change in PCCT match w.r.t. the target EID kernel.

Abbreviations: EID, Energy integrating detectors; IR, Iterative reconstruction; PCCT, Photon‐counting computed tomography.

**FIGURE 6:**
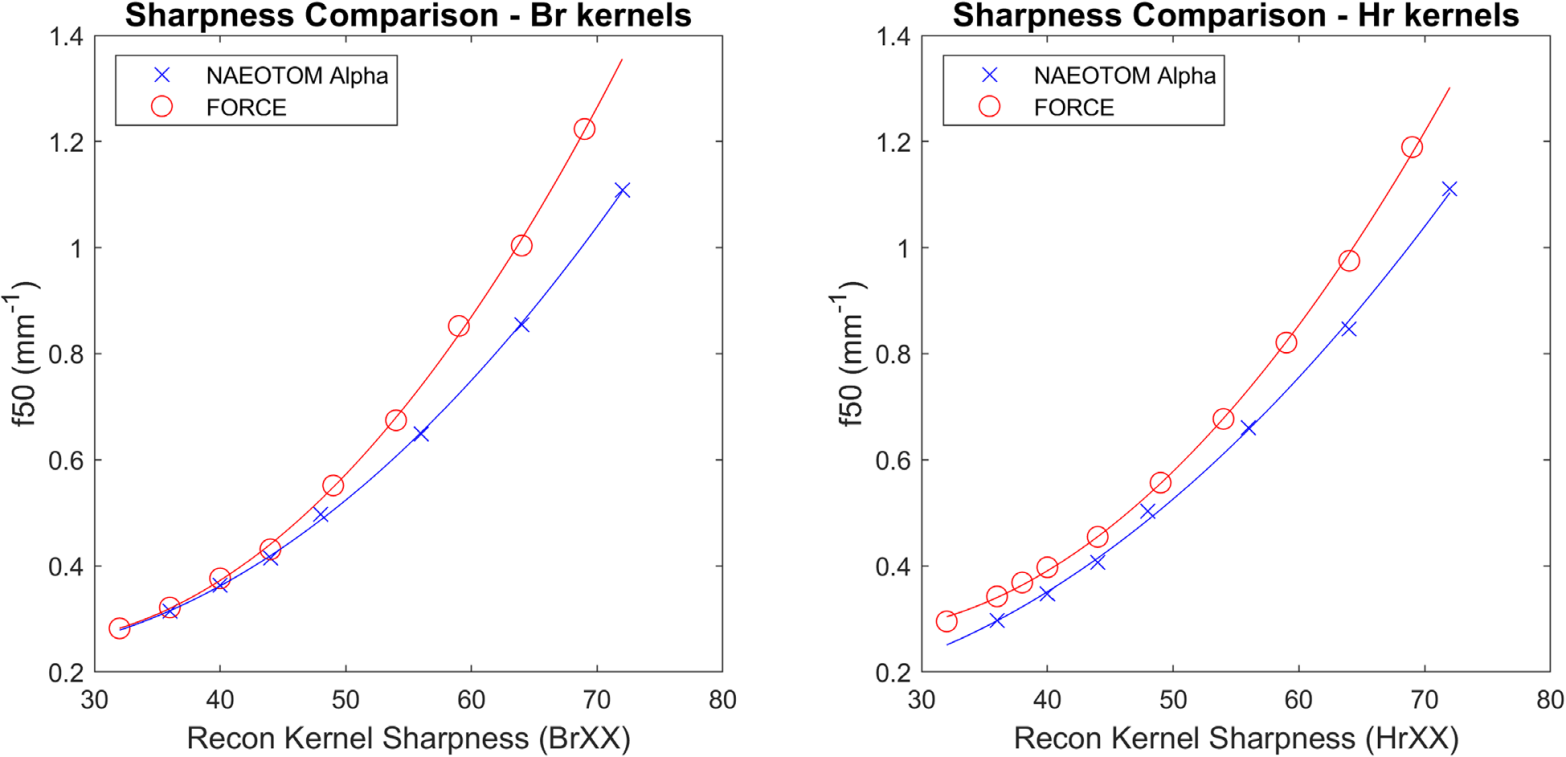
Comparison between indicated reconstruction kernel sharpness and measured spatial resolution with Body regular (Br, Right) and Head regular (Hr, Left) kernel families for EID and PCCT reconstructions. All data are filtered back‐projection reconstructions.EID, Energy integrating detectors; IR, Iterative reconstruction; PCCT, Photon‐counting computed tomography.

For “Body regular” (Br) and “Head regular” (Hr) reconstruction families results show that the EID reconstructions had better spatial resolution in terms of f50 than PCCT reconstructions for matched reconstruction kernel names, as shown in Figure 6.

### 3.3 IR characterization

**FIGURE 7:**
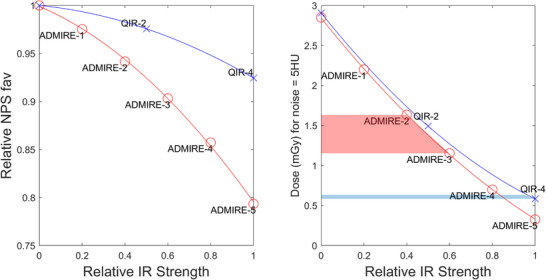
Average noise power spectrum frequency relative to filtered back‐projection (Left) and relative dose versus IR strength for EID (“ADMIRE”) and PCCT (“QIR”) implementations of IR (Right). ADMIRE, Advanced Modeled Iterative Reconstruction; EID, Energy integrating detectors; IR, Iterative reconstruction; PCCT, Photon‐counting computed tomography; QIR, Quantum Iterative Reconstruction.

Figure 7 shows the noise texture, *f_av_*, relative to filtered back‐projection (FBP) as a function of IR strength for the Br40 kernel. For the EID system, *f_av_* dropped to 80% of FBP level with IR strength set to maximum, while for the PCCT system fav dropped to 93% of FBP level. To compare the ability of IR to reduce noise magnitude for each scanner type, Figure 7 shows radiation dose (CTDI_vol_) in mGy required to obtain a noise magnitude of 5HU as a function of iterative strength.

## 4 DISCUSSION

The algorithm described in this work—in short, identifying an existing EID reconstruction kernel and IR strength and subsequently finding the PCCT kernel/IR that most closely matches the noise texture, matches or improves spatial resolution, and matches or reduces dose—represents only the initial phase of bringing a PCCT system into routine clinical use. One of the primary benefits of this particular PCCT technology is the availability of spectral information for all exams, allowing—amongst other post‐processing—the production of virtual monoenergetic image sets (VMIs). For this work, only 70 keV image sets were evaluated, as this is approximately the effective energy of the 120 kVp acquisition spectrum and is the default non‐IV‐contrast enhanced VMI energy value for this system. In clinical practice, the VMI keV value should be tailored to the clinical task being evaluated by the exam. For example, the default IV‐contrast enhanced VMI keV value of the system is 55 keV, and our institution has had success with reconstructions down to 40 keV for maximizing iodinated contrast enhancement. In anticipation of utilizing the PCCT system for more quantitative imaging tasks, VMI datasets were used for this work. An alternative to VMIs for this particular system is the production of low‐energy threshold “T3D” image sets that utilize the benefits of removing electronic noise and weighting low and high energy photons equally, resulting in more traditional EID‐like images acquired at the same acquisition kV. Different post‐processing between VMI and T3D images, including beam hardening corrections, may result in image perception metrics that do not agree with the findings of this study. Further work is required to confirm that the matching paradigm used in this work would hold for other keV values or T3D image sets.

In general, the results presented here point to a straightforward methodology for translating existing EID‐based kernels and reconstruction strengths to PCCT reconstruction parameters using common metrology and framework: increase the PCCT kernel sharpness one level from existing EID setting, while increasing the level of IR strength by 1−2 steps provide equivalent image quality performance if so desired.

Highlighted on Figure 7 are the potential dose savings between using ADMIRE strengths 2−3 on the EID scanner compared using QIR strength 4 on PCCT, which are the respective IR strengths where noise texture is within 10% of FBP level. However, although this suggests substantial dose reduction for routine clinical work—in some cases 20%−50% reduced dose for our institutions reoutine kernels it is recommended to match or only slightly reduce clinical doses when incorporating a PCCT into an existing EID fleet.

Matching or improving spatial resolution while simultaneously matching noise texture is an appropriate starting point, but other technical parameters including keV, associated window/level settings, and slice thickness should be evaluated with appropriate radiologist and physicist feedback before realizing the full dose reduction potential. In addition, all dose reduction potential described in this work is in terms of noise magnitude, which is not the sole metric to be used to guide dose decisions in terms of detectability and overall diagnostic accuracy.^25^

All analysis reported in this work was performed on the syngo CT VA40A software version. Starting with VA40A_SP1, the QIR strengths were adjusted such VA40A QIR strength x was equal to VA40A_SP1 QIR strength x‐1. Because of this update, QIR results from this work should be shifted down by one strength “level” for systems running the syngo CT VA40_SP1 software version or later.

Finally, this work does not address the next step in incorporating the PCCT into clinical use, which is to identify where diagnostic quality may be improved with the system. This includes the possibility of ultra‐high‐resolution mode scanning with very thin 0.2 mm slices, producing multiple VMI data sets to improve intravenous iodinated contrast visualization (low keV) or reducing metal artifacts (high keV), or trading dose reduction for drastically improved spatial resolution at matched noise magnitude. These are all topics for future work.

## 5 CONCLUSION

Incorporating a PCCT system into an existing fleet of EID‐based CT scanners is a multi‐step process. This work has identified a methodology for translating image perception from existing systems to a PCCT by identifying matching reconstruction kernels and IR strengths. By appropriately weighting and matching image perception metric between systems, an initial protocol design approach was outlined. In addition, potential for dose reduction due to inherent properties of the PCCT system and improved IR performance was demonstrated.

## AUTHOR CONTRIBUTIONS

Steven T. Bache provided substantial contributions to the conception or design of the work, the acquisition, analysis, and interpretation of data for the work, drafting the work or revising it critically for important intellectual content, final approval of the version to be published, and agreement to be accountable for all aspects of the work in ensuring that questions related to the accuracy or integrity of any part of the work are appropriately investigated and resolved. Ehsan Samei provided substantial contributions to the conception or design of the work, revising it critically for important intellectual content, final approval of the version to be published, and agreement to be accountable for all aspects of the work in ensuring that questions related to the accuracy or integrity of any part of the work are appropriately investigated and resolved.

## CONFLICT OF INTEREST STATEMENT

The authors declare no conflicts of interest.

## Supporting information

Supporting Information

Supporting Information

Supporting Information

Supporting Information
