# Supplementary figures and images for: A methodology for incorporating a photon‐counting CT system into routine clinical use
Source: J Appl Clin Med Phys. 2023 Jun 30;24(8):e14069. doi: 10.1002/acm2.14069 (PMC10402682; doi:10.1002/acm2.14069)

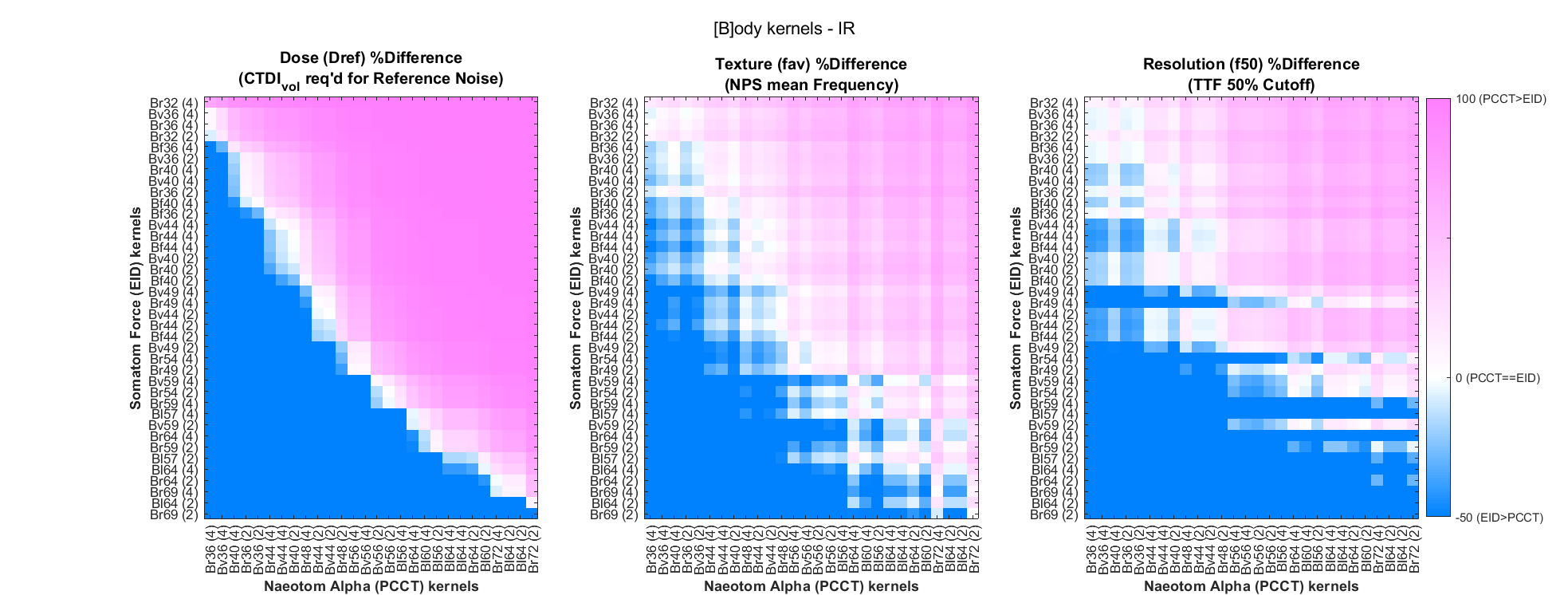

Supplement: Supplementary file 1 — Supporting Information [file ACM2-24-e14069-s001.tif]

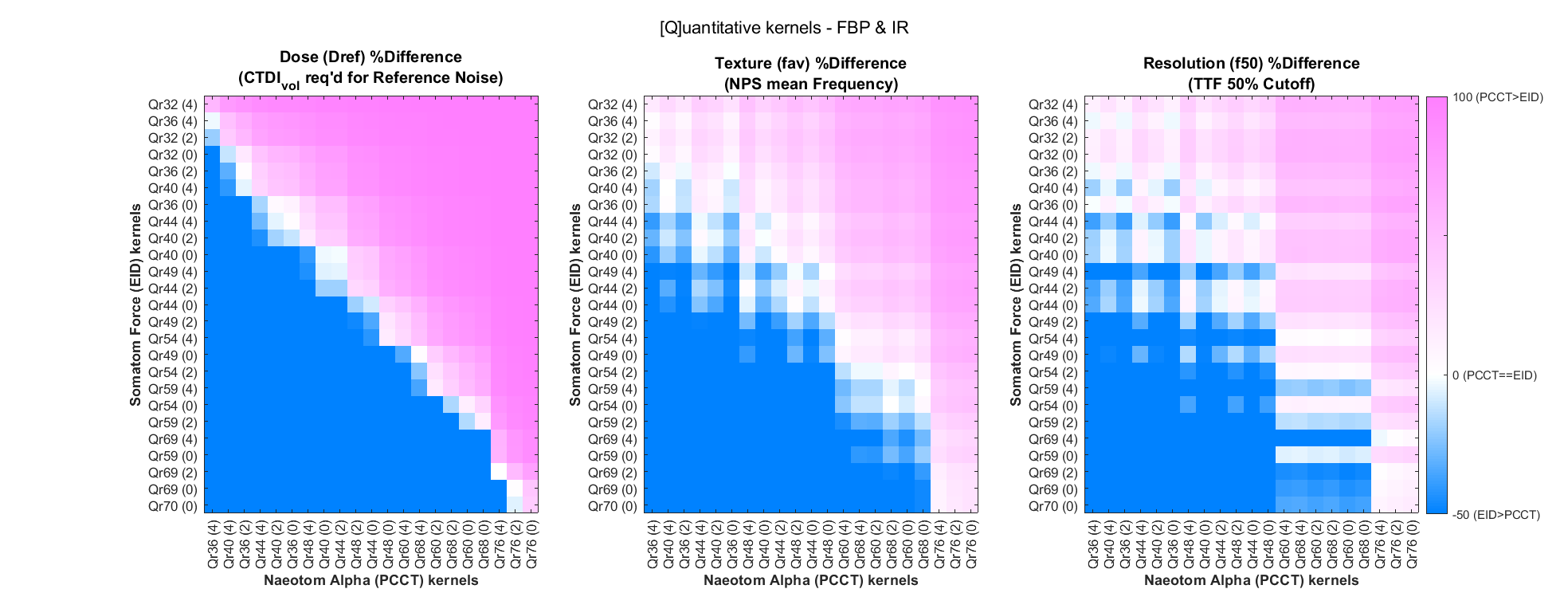

Supplement: Supplementary file 2 — Supporting Information [file ACM2-24-e14069-s004.tif]

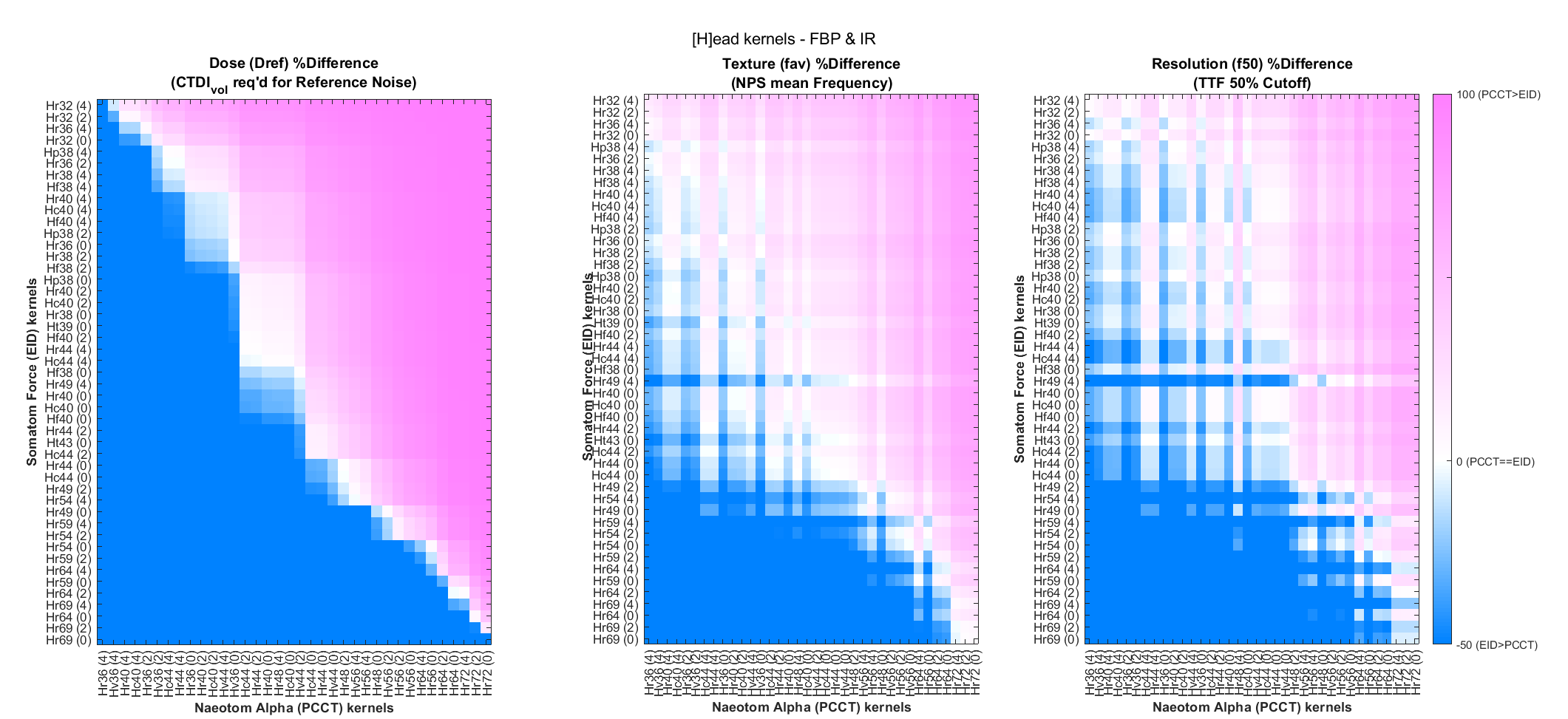

Supplement: Supplementary file 3 — Supporting Information [file ACM2-24-e14069-s002.tif]

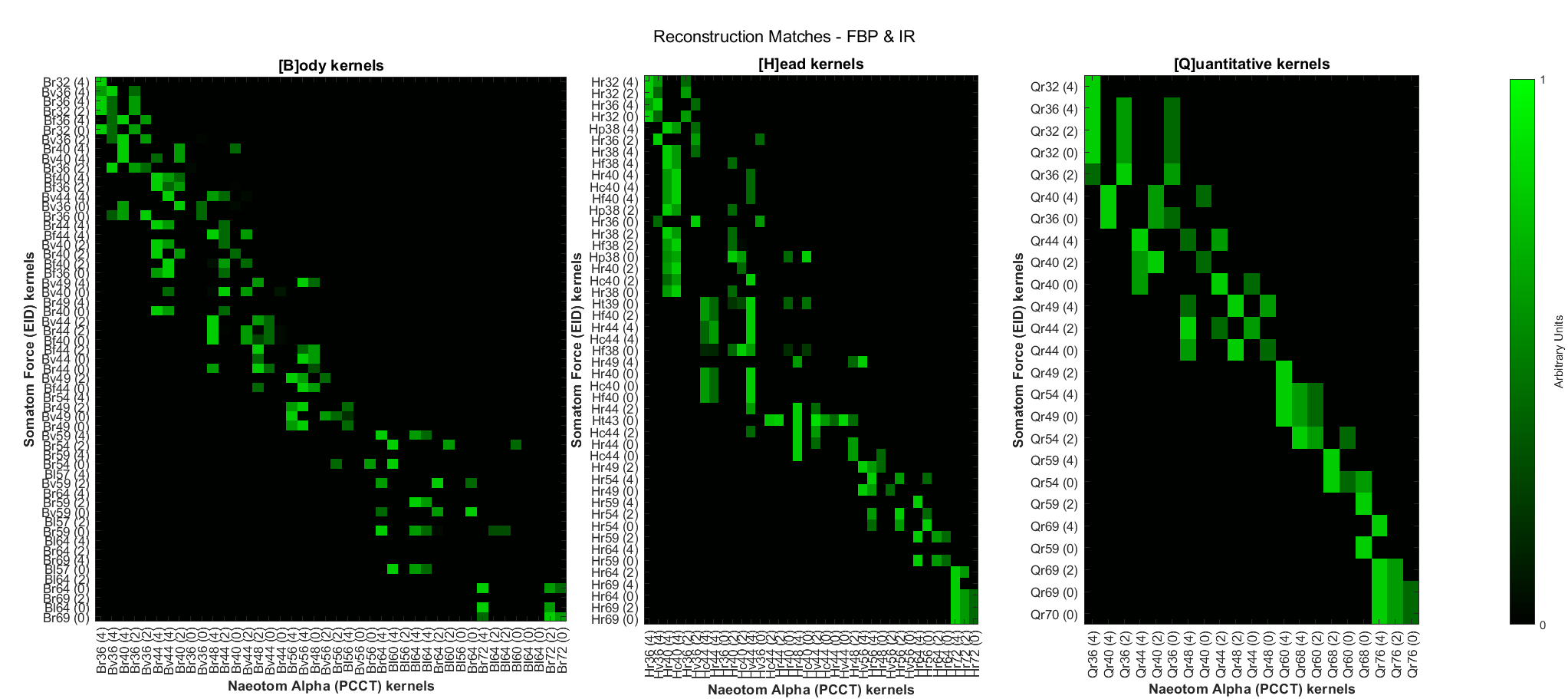

Supplement: Supplementary file 4 — Supporting Information [file ACM2-24-e14069-s003.tif]
